# The unsteady state and inertia of chemical regulation under the US Toxic Substances Control Act

**DOI:** 10.1371/journal.pbio.2002404

**Published:** 2017-12-18

**Authors:** Sheldon Krimsky

**Affiliations:** Tufts University, Medford, Massachusetts, United States of America; National Institute of Environmental Health Sciences, United States of America

## Abstract

After 40 years, the 1976 US Toxic Substances Control Act (TSCA) was revised under the Frank R. Lautenberg Chemical Safety for the 21st Century Act. Its original goals of protecting the public from hazardous chemicals were hindered by complex and cumbersome administrative burdens, data limitations, vulnerabilities in risk assessments, and recurring corporate lawsuits. As a result, countless chemicals were entered into commercial use without toxicological information. Few chemicals of the many identified as potential public health threats were regulated or banned. This paper explores the factors that have worked against a comprehensive and rational policy for regulating toxic chemicals and discusses whether the TSCA revisions offer greater public protection against existing and new chemicals.

This Essay is part of the *Challenges in Environmental Health: Closing the Gap between Evidence and Regulations Collection*.

## Introduction

The phrase “promote the general welfare” appears in 2 places in the US Constitution: the Preamble and Article 1, Section 8. This vague, but weighty, dictum gives Congress the grounds for collecting taxes and expending funds for passing public health laws to protect people at work, home, and in the environment from dangerous products, working conditions, and industrial pollutants. Congress has enacted laws that have afforded the Executive Branch the authority and responsibility to assess risks that threaten the “general welfare” and commensurate with its findings, and establish regulations or guidelines for reducing the hazards under the aegis of public health protection. Congress has regulated dangerous substances in the environment using the General Welfare clause and the Public Goods doctrine in which dangerous substances cross interstate public lands and waterways.

The first food and drug legislation, which was passed in 1906, prohibited interstate commerce in misbranded and adulterated foods, drinks, and drugs. Protecting public health was largely about accurate labeling and contamination. It was assumed that people were wise enough to avoid products with dangerous contaminants like arsenic once they were appropriately labeled. Most dangerous exposures came from burning, eating, or handling dangerous substances found in nature. The dangers to public health rose exponentially with the advances in synthetic organic chemistry during the 20th century as new untested compounds were introduced into commerce.

The government’s role was to act when there was a public health crisis, as when consumers were poisoned by contaminated food. Food and drug manufacturers had the responsibility for producing and selling safe products. It was the government’s burden to demonstrate that a commercial product was unsafe—until 107 people died from a poisonous ingredient in the drug Elixir Sulfanilamide. The 1937 incident hastened passage of the Federal Food Drug and Cosmetic Act the next year. The law reversed the burden of proof for drugs by requiring manufacturers to provide scientific proof that new products could be safely used before putting them on the market. The law (which also regulated cosmetics and therapeutic devices) also regulated toxic residues on foods.

After World War II, commercial production of industrial chemicals exploded, yet remained largely unregulated as they became ubiquitous in agriculture, manufacturing, mining, construction, and consumer products. The first major piece of legislation regulating industrial chemicals (other than food additives, pharmaceuticals, and pesticides), enacted in 1976 under the Toxic Substances Control Act (TSCA), was ostensibly a chemical notification act, and shall be explained in a later section. Throughout this article, the 1976 TSCA act will be referred to as “original TSCA.” The first major revision to TSCA, under The Frank R. Lautenberg Chemical Safety for the 21st Century Act, was passed on June 22, 2016, which will be referred to as the “revised TSCA.”

This article shall address past and current legislative approaches to regulating existing and new chemicals through both the original and revised TSCA laws, and shall discuss the factors that have impeded an aggressive health and safety approach to industrial chemicals in the United States. The factors that have limited a comprehensive and rational approach to chemical safety include the following: a government burden to demonstrate “unreasonable risk” rather than a manufacturer burden to demonstrate that a chemical entering commerce is safe; limited federal authority to require health and safety data; lack of agency capacity to analyze large amounts of data for thousands of chemicals in a timely manner; procedural complexity for implementing the law, inconclusiveness of data, or failure of replication in testing outcomes resulting in regulatory stalemate; and finally, delays arising from corporate legal challenges that frequently follow new chemical safety rules.

## Starting position for new chemicals

There are 2 fundamental ways to address the risks of a new substance (or technology): Thesis 1 assumes the substance is unsafe, unless it can be proven safe, and Thesis 2 assumes the substance is safe, unless it can be proven unsafe.

A health and safety regulatory system operating from Thesis 1 would not accept a substance into commerce unless and until it could demonstrate that the substance was safe. In other words, the statement “substance X is unsafe” has to be disproven by relevant evidence. There is no obvious way to prove a substance is safe, or alternatively to show that the statement “substance X is unsafe” is false. Philosopher Karl Popper showed that induction is a faulty method of fixing belief and that a deductive methodology offers a more dependable epistemology [1]. To demonstrate “substance X is safe” by appealing to instances where it is safe does not allow us to conclude that the next instance may prove it safe. Falsification of “substance X is safe” requires one counter instance that it is unsafe. The best that can be done is to test the substance through a variety of plausible worst-case scenarios and show that every effort to demonstrate “substance X is unsafe” fails. Failing to demonstrate that “substance X is unsafe” allows us to infer that “substance X is safe.”

In cases in which we do not have conclusive but only preliminary evidence that a substance is unsafe, Thesis 1 implies that we continue to assume it is unsafe unless it is conclusively proven otherwise by the negative outcomes of worst-case scenarios. This is a form of the Precautionary Principle, whereby a substance suspected of causing harm, is not approved for commerce until the suspicion can be eliminated.

US federal drug laws operate largely according to Thesis 1. Manufacturers must demonstrate to the Food and Drug Administration (FDA) through animal and human studies that the drug is safe (1938 law) and effective (1962 law) before it can be licensed. Modern pesticide laws also require toxicity testing prior to the introduction of an insecticide, fungicide, or herbicide into commerce in order to determine whether they pose unintended or unreasonable risks to humans, animals, and the environment. Drugs and pesticides incorporate the idea that first we test, then we determine whether the product meets a safety standard.

Thesis 2 has been applied in several forms to US regulatory policy. Under the “generally regarded as safe before we test it” criteria, a substance is assumed safe for commercial use unless shown otherwise. The Food Additives Amendment of 1958 established a list of 700 food additives that were exempt from the extensive testing the law required of manufacturers before introducing new food additives into commerce. These substances were designated as generally regarded as safe (GRAS). Current FDA rules allow food manufacturers to submit food additives they affirm as satisfying GRAS criteria from their own contracted studies [2].

Many existing chemicals have been given a GRAS-like designation under the original TSCA. Also, new chemicals that have not been subjected to toxicological testing have inherited this moniker once they are accepted into commerce. The provisions of the original TSCA are largely premised on Thesis 2. As we shall see, the revised TSCA moves slightly toward Thesis 1 but at a very slow pace and bearing many of the impediments of the original TSCA.

In an ideal world where there is no limit on human resources, funds, or time within which information can be collected, analyzed, and decisions made, Thesis 1 would be preferable and most protective of human health. In other words, a new chemical would not be approved for commercial use without a complete toxicological profile and characterization of potential hazards.

## Original TSCA and the burden of proof for chemical safety

After the original TSCA was passed in 1976, the Environmental Protection Agency (EPA) compiled an inventory of 62,000 industrial chemicals then in use. These chemicals were essentially grandfathered into commercial use and assumed to be safe unless EPA could show otherwise [3]. EPA could, in theory, take an existing chemical off the market but it would have to meet a formidable burden, namely produce substantial evidence that the chemical presents or will present an unreasonable risk to health and the environment. The concept of “unreasonable risk” was not defined in the legislation or in subsequent regulations, and this ambiguity opened up judicial challenges to agency findings. In addition, under the act, EPA had to demonstrate that the benefits of regulating a chemical were greater than the costs—to the manufacturer, to companies utilizing the chemical, and to the economy—and that its regulation offered greater benefit than the social value of the products it was used to create. Finally, the agency had to demonstrate that it chose the least burdensome method for reducing or eliminating unreasonable risk [3].

Under the original TSCA, there were separate criteria for introducing new chemicals into commerce. A new chemical is one that is not on the TSCA inventory and therefore is not already in commerce. But this too placed a great burden on the agency. Chemical manufacturers had to notify the EPA by submitting a premanufacture notice (PMN) before marketing a new chemical. The PMN, however, did not require that a company produce a minimum amount of health and safety data. After manufacturers submitted a PMN to the EPA, the agency had 90 days to determine whether it was unsafe or allow its use. There were no penalties associated with lack of data. In 2003 the EPA found that 85% of the PMNs lacked data on health effects [4]. Wilson and Schwarzman noted: “Hindered by limited data and the short time permitted for the agency’s review, the EPA has taken some form of action on <10% of the 36,000 chemicals that producers have proposed for commercial use between 1979 and 2004” [3].

Added to the deficiency of data, the EPA’s resources have not come close to meeting the statutory responsibilities of the agency, even under Thesis 2-type regulation. The Government Accountability Office (GAO) reported in 2013 [5] that since 1976, the EPA has used its authority under TSCA to limit or ban only 5 existing chemicals: fully halogenated chlorofluoroalkanes, polychlorinated biphenyls (PCBs), dioxin, asbestos (later overturned by the courts) [6], and hexavalent chromium. The agency did not have sufficient information to declare any other of the tens of thousands of chemicals unsafe. The original TSCA did not give the EPA authority to continually review the safety of a chemical once it was introduced into commerce [5].

In 2010 the Office of Inspector General (OIG) reviewed the EPA’s New Chemical Program and found that it “is limited by an absence of test data and a reliance on modeling because TSCA does not require upfront testing as part of a Premanufacture Notice (PMN) submission…the majority of PMN submissions do not include chemical toxicity or environmental fate data [7].” The OIG found that between 1996 and 2008, the EPA received approximately 1,500 PMNs annually, and on average less than 10% were subject to regulatory action.

With an average of 700 chemicals added each year, the EPA would have approximately 85,000 chemicals on the TSCA inventory by 2017. Estimates that there are within 10 million chemicals in existence is an order of magnitude lower than the 100 million unique chemicals that make up the Chemical Abstract Service (CAS) Registry of the American Chemical Society [8]. Thus, about one-thousandth of the number of discrete chemicals in the CAS Registry has been entered into commerce. In order to make some headway in undertaking a risk assessment for the estimated 85,000 chemicals in commerce, the agency has little recourse but to prioritize its efforts by choosing a subset of chemicals based on preliminary toxicological information, production volume, and human exposure [4].

Because the original TSCA gave the EPA little authority to require toxicological information from manufacturers of new and old chemicals, the agency could not possibly have fulfilled even a priority setting program based on a complete review of existing chemicals. The bar for meeting both the unreasonable risk standard and for acquiring data was quite high, which limited EPA’s effectiveness even without the other constraints including human and economic resources to conduct its work.

The Government’s finite resources to investigate chemicals and enforce its decisions are further stretched by industry lawsuits that challenge regulatory decisions with major economic impacts, thereby restricting the EPA to regulate no more than 2–3 chemicals per year [5].

According to the GAO) report of 2013 [5], under the original TSCA it could take about 5 years for the EPA to issue a test rule for a chemical and another 2–2.5-years for companies to provide the data. In addition, most of the toxicity data the EPA obtains from industry is considered “screening level” information and can only be used to identify potential hazards. That data cannot be used to establish that a chemical poses an unreasonable risk to human health [5]. These are some of the reasons it took decades to regulate a single toxic chemical.

In 1994, the GAO recommended that Congress strengthen the EPA’s authority to obtain chemical toxicity information from companies for both new chemicals and existing chemicals on the TSCA inventory [9]. The GAO testified that after 18 years, “TSCA has not played a major role in EPA’s efforts to protect human health and the environment from the harmful effects of toxic chemicals” [9]. The congressional impetus to revise TSCA was still years away.

## Easing regulatory burdens and new mandates in revised TSCA

As criticisms of US chemical policy mounted and the Registration, Evaluation, Authorization, and Restriction of Chemicals (REACH) program passed by the European Parliament in 2006 set a new standard for chemical safety, multi-stakeholder efforts to revise TSCA with high expectations began to gain traction. Senator Frank Lautenberg (D-NJ) introduced The Safe Chemicals Act in 2010 (S.3209), which environmentalists considered the most progressive of the bills to date as it introduced the concept of “negligible risk” and “reasonable certainty of no harm,” giving the EPA a higher standard of safety for rulemaking than unreasonable risk. The bill mandated EPA to review 300 chemicals at any given time until all the chemicals had a safety determination. And it excluded judicial review once the EPA Administrator determined that a manufacturer or processor had not established that the chemical meets the safety standard.

The revised TSCA was passed on June 22, 2016, and while not reaching the high protective standards set in Lautenberg’s original bill, it did gain bi-partisan support. The revised TSCA gave the EPA somewhat greater authority than the original TSCA to require chemical companies to provide the agency with chemical toxicity and exposure data to enable the agency to do a hazard evaluation.

The new authorities under the EPA include the power to determine unreasonable risk to health without having to trade it away for costs and other non-risk factors, as in a cost-benefit analysis; attention to vulnerable populations in risk determinations; once a determination of unreasonable risk is made, restrictions of the chemical must be made; deadlines set for all risk evaluations; from existing chemicals on the TSCA registry, the EPA must undertake required risk evaluations for each chemical it designates as a high-priority substance; companies must pay either 100% or 50% of the cost for evaluations of chemicals they request or that are on the EPA’s Work Plan, respectively; the EPA is mandated to review and make a risk determination for all new chemicals; and the agency must make an affirmative finding about a new chemical before it can enter commerce.

Congress removed the requirement that the EPA use the least burdensome approach to regulation. It also gave the agency new authority to require testing of a chemical before it promulgated a rule. It mandated that the EPA review every new chemical or significant new use of an existing chemical to determine whether it presented an unreasonable risk and gave the EPA authority to apply PMN requirements to existing chemicals if it finds there is a reasonable potential for exposure.

Preemption of state regulatory activities for chemicals was part of the original TSCA and is part of the revised TSCA. In the original TSCA, states could get a waiver for preemption if they offered a significantly higher degree of protection from chemical risks than that of the federal government. The revisions created limited preemption of state chemical regulations. For example, states would not be allowed to restrict a chemical that the EPA found as not presenting an unreasonable risk. Also, new state chemical regulations will be preempted, while the EPA is undertaking a safety assessment of a high-priority chemical.

Notwithstanding the EPA’s new authority and its mandates under the revised TSCA, the prospect of making headway in assessing tens of thousands of chemicals currently in use remains daunting. The agency faces considerable hurdles in data acquisition and building the requisite human resources to analyze the massive amounts of data. It must prepare for challenges to its determination of unreasonable risk (a middle ground between absolute safety and actual harm), a term currently ill-defined and thus subject to different interpretations. And the EPA must build a response capability to what will inevitably be challenges to its risk assessment and rulemaking.

Fulfilling the revised TSCA’s mandates could take many years as the EPA would have to contract out or require companies to develop new tests to meet its risk assessment data needs. Without this basic information, the EPA cannot prioritize the tens of thousands of chemicals in commerce for a determination of relative risks. A Republican President and a Republican majority in Congress have leaned heavily toward diminishing the authority of the EPA, which means reducing its resources to carry out regulatory programs. Past experience shows that legal authority without the requisite resources is a recipe for inaction or glacial progress in public health regulations.

Any comprehensive and rational approach to chemical regulation has failed largely because of the cost of good data and the expense of litigation. In Applegate’s words: “Faced with huge data gaps and uncertainties agency choices are necessarily driven by policy and not by ‘fact’” [10].

## Toxicological studies

Among the factors that have impeded regulatory action on chemicals is the time it takes to validate methods for acquiring health and safety data and the challenges of evaluating findings that do not lend themselves to traditional toxicology methods. Such factors have been especially problematic in the studies of low-dose exposures. Among the recurring questions are: What is meant by low dose and how should low-dose effects be tested?

Human effects of extremely low doses of a toxic chemical challenge the traditional toxicology dictum “the dose makes the poison,” which assumes that a substance does not cause harm below a dose that elicits no effect. But for some chemicals, including endocrine-disrupting compounds, effects are found at low, but not high or intermediate, doses. These chemicals do not produce a linear dose-response relationship but what’s called a nonmonotonic dose response relationship (NMDR), which is characterized by a curve whose slope changes direction within the range of tested doses. As a result, standard toxicology methods for human safety by extrapolating from high-dose testing may not be appropriate. What is important to understand is that different mechanisms may be operating at low and high doses, but they may be manifesting as a common phenotype [11]. The chemicals must be tested over a larger dose range, thus lengthening the time period of the risk assessment.

The timing of exposure can also influence outcomes. If low doses of a chemical are tested only on adult nonpregnant animals, the effects of those doses on sensitive windows of development, [12] such as the embryo, may not be revealed. And potential adverse effects of a chemical may not be apparent unless it’s tested at a range of doses in embryos and adults for different endpoints, including carcinogenicity, mutagenicity, immunotoxicity, genotoxicity, neurotoxicity, and endocrine disruption, both in adult and in utero studies.

There are ongoing debates over the validation and replication of low-dose studies of endocrine disruptors [13]. Without consensus within the scientific community on the validation of toxicology studies, rulemaking for the chemical usually does not proceed without years of re-testing. After scientists found low-dose effects on prostate development of male rodents from exposure to endocrine disruptors such as bisphenol A (BPA) and diethylstilbestrol (DES) [14], and replicated the results [15], industry-funded experiments failed to find similar effects [16,17]. Vom Saal and Hughes commented that as of December 2004, 94 out of 115 published in vivo studies showed significant low-dose effects of BPA, but no industry studies report such effects [18]. Detection of low-dose effects in animal tests requires exquisite sophistication, often involving different strains of mice, using different brands of animal feed. Writing about the failure of replication, Sheehan wrote, “there are numerous ways that complex experiments can fail but often only a few ways that they can succeed” [13].

There is no clear and unambiguous criterion of unreasonable risk, and there are a lot of obstacles in reaching scientific consensus given the modeling that must be done, including the extrapolation from animal studies to human effects, and the replication of results. For example, in 2002, the National Toxicology Program’s report on low dose acknowledged that several studies provide credible evidence for low-does effects of BPA at 2–20 μg/kg per day, but the experiments were not replicated, thus undermining regulation [19]. When the EPA has to navigate through the bramble bush of contested science, it has to prepare for litigious battles.

Since 1996, the EPA has the authority to screen certain chemicals for endocrine disrupting properties (e.g., pesticides under The Food Quality Protection Act (FQPA) [20] and contaminants under the Safe Drinking Water Act [21]). Recommendations for how the agency should do so were issued by the Endocrine Disruptor Screening and Testing Advisory Committee (EDSTAC) in 1998. The EDSTAC report recommended “high throughput screening of 15,000 chemicals currently produced in an amount equal to or greater than 10,000 lbs. per year,” including TSCA inventory chemicals [22]. The FQPA expected the EPA to complete the screening by the year 2000, an unrealistic expectation given the administrative, technical, and political challenges. The EPA issued 2 lists of chemicals for endocrine disruptor Tier 1 screening: List 1 consists of 67 pesticide chemicals, with 15 voluntarily withdrawn, leaving 52 for which Tier 1 screening was completed, and 18 of those were recommended for Tier 2 screening. List 2 consists of 109 chemicals, 41 pesticide active ingredients, and 68 chemicals identified under the Safe Drinking Water Act.

Vandenberg et al. have sought to break the stalemate for gaining consensus over the assessment of endocrine disruptors by developing a framework and methodology for systematic review of the literature [12]. Even so, there are no agreed-upon validated in vitro assays for a substantial number of endocrine effects [23], although there has been considerable progress in validated assays for estrogenic and androgenic pathways [24,25]. International harmonization of validated assays for endocrine disruptors is still in its infancy [26].

This case illustrates just one of the many areas in which uncertainty enters a chemical risk analysis that can delay or derail rulemaking [27].

## Discussion: Future prospects under revised TSCA

The TSCA revisions have addressed some of the obstacles to obtaining toxicological data and setting risk standards for chemicals by establishing clearer time tables, such as a 3.5-year period for establishing a list of 20 high-profile chemicals that must undergo evaluation. They have also reduced some of the procedural obstacles that have limited the EPA from requiring testing of chemicals and acquiring data held by the manufacturers. Ironically, it also adds administrative burdens on the EPA such as meeting time tables and requiring the agency to make a risk determination for all new chemicals.

To what extent will the new law enable the EPA to catch up for the 40 years that chemicals were allowed into commerce without sufficient data on health and safety? How will it meet greater responsibilities without the requisite resources? How will it address the litigation that typically accompanies more aggressive regulatory policy? It is useful to recall what EDSTAC wrote about the limits on the original TSCA in 1998: “Although EPA has authority to order testing of chemicals under TSCA, in the nearly 20 years of TSCA’s existence, the authority has been used for only 121 chemicals. This is not an indication of how much more information might really be needed but, rather, the administrative challenges of mounting an information request” [22].

The revised TSCA eases some of the past regulatory burdens, such as requiring that the rule for regulating a chemical undergo a strict cost-benefit analysis, but still requires an analysis of the costs, benefits, and cost-effectiveness of its proposed regulatory action. These ancillary requirements could be subject to litigation on the challenge that they are inaccurate.

The ultimate effectiveness of the revised TSCA in reviewing the chemicals in current use depends on the number of high-priority versus low-priority chemicals on the TSCA inventory of more than 85,000 chemicals that can be evaluated over a reasonable time period. The law requires the EPA to have 10 ongoing risk evaluations in the first 180 days and 20 within 3.5 years. Let us assume it will have to undertake risk evaluations for 10% of the existing chemicals—that’s 8,500 in groups of 20 to be completed every 3.5 years. That would take about 1,500 years to complete. That is not a very encouraging outcome and mirrors the glacial pace of evaluating endocrine-disrupting chemicals. With a priority list of 500 chemicals a year and a 3-year completion time, the task could be completed in 50 years.

Without greatly increasing the agency’s resources [28], it is unlikely that it can meet its mandate even as modest as it is in the revised TSCA. “The scarcity of regulatory resources therefore mirrors the scarcity of information” [10]. Since the law was passed, the backlog of meeting industry requests for new chemicals has doubled from over 300 to over 600 requests, largely because the EPA can demand toxicity data from the industry. At this pace, pressure will grow from the industry to speed up the pace of allowing new chemicals on the market, which has historically resulted in 600–1,000 chemicals a year. With an antiregulatory administration, it is unlikely that a slowdown in the approval of new chemicals would be acceptable. The Trump Administration proposes to reduce the EPA budget by 31%, cutting the current $8.1 billion budget by $2.6 billion [29], which could set chemical protection back 40 years, although Congress’s proposed cuts for 2018 are significantly lower.

Among the most critical challenges and obstacles to TSCA, original and revised, is the unreasonable risk standard. Applegate summed it up as follows:

“…the unreasonable risk standard has been a failure. It has imposed huge information demands, invited contention and judicial intervention, and thwarted regulatory action. A risk standard that tries to measure a complex and incompletely understood human health effect like cancer, that considers cost and other factors, that must do all this for each chemical that it wishes to control—and must do it with relative precision—makes enormous information and resource demands on the regulator. Where the agency has the burden of proof, the uncertainty of all these elements leads to regulatory paralysis, especially in the face of a well-framed opposition and critical courts” [10].

Is there a solution to this finding? Perhaps. Returning to the 2 theses introduced earlier, it may help to remind ourselves that operating under the assumption that a substance is safe until proven otherwise, it has taken 20–25 years after a chemical has entered the commercial market to strictly regulate or prohibit its use, as in the case of lead, PCBs, asbestos, and dichlorodiphenyltrichloroethane (DDT). Manufacturers challenge the science and use the uncertainties in the risk assessment opportunistically to demand further studies until there is unimpeachable consensus that the chemical is a public health hazard. If we operated under the assumption that a substance is unsafe until proven otherwise, the onus would be reversed: the manufacturers would have to spend decades in research to remove all uncertainty and demonstrate that a chemical was unimpeachably safe—a true precautionary approach. Society would not be spending a millennium playing catch-up with the unknown risks from having adopted an approach that favors commerce over health.

## Abbreviations

BPA: bisphenol A
CAS: Chemical Abstract Service
DDT: dichlorodiphenyltrichloroethane
DES: diethylstilbestrol
EDSTAC: Endocrine Disruptor Screening and Testing Advisory Committee
FDA: Food and Drug Administration
FQPA: Food Quality Protection Act
GAO: Government Accountability Office
GRAS: generally regarded as safe
NMDR: nonmonotonoic dose response relationship
OIG: Office of Inspector General
PCB: polychlorinated biphenyl
PMN: premanufacture notice
REACH: Registration, Evaluation, Authorization, and Restriction of Chemicals
TSCA: Toxic Substances Control Act
